# Phosphorylated IκBα Predicts Poor Prognosis in Activated B-Cell Lymphoma and Its Inhibition with Thymoquinone Induces Apoptosis via ROS Release

**DOI:** 10.1371/journal.pone.0060540

**Published:** 2013-03-28

**Authors:** Azhar R. Hussain, Shahab Uddin, Maqbool Ahmed, Fouad Al-Dayel, Prashant P. Bavi, Khawla S. Al-Kuraya

**Affiliations:** 1 Human Cancer Genomic Research, Research Center, King Faisal Specialist Hospital and Research Center, Riyadh, Saudi Arabia; 2 Department of Pathology, King Faisal Specialist Hospital and Research Center, Riyadh, Saudi Arabia; 3 Al-Faisal University, Riyadh, Saudi Arabia; University of Navarra, Center for Applied Medical Research, Spain

## Abstract

Activated B-cell lymphoma (ABC), one of the three subtypes of Diffuse Large B-cell Lymphoma (DLBCL) has the worst survival rate after upfront chemotherapy and is characterized by constitutively activated NFκB. We therefore studied the role of NFκB In a cohort of clinical DLBCL samples and ABC cell lines. In our clinical tissue microarray cohort of DLBCL samples, p-IκBα was detected in 38.3% of ABC DLBCL and was an independent prognostic marker for poor survival. *In vitro*, we found that Thymoquinone (TQ), a natural compound isolated from *Nigella sativa* caused release of ROS in ABC cells. TQ-mediated release of ROS in turn inhibited NFκB activity by dephosphorylating IκBα and decreased translocation of p65 subunit of NFκB in the nuclear compartment in ABC cell lines. This led to inhibition of cell viability and induction of mitochondrial dependent apoptosis in ABC-DLBCL cell lines. Additionally, TQ treatment also caused up-regulation of death receptor 5 (DR5), however, up-regulation of DR5 did not play a role in TQ-induced apoptosis. Finally, combination of sub-optimal doses of TQ and TRAIL induced efficient apoptosis in ABC-DLBCL cell lines. These data show that p-IκBα can be used as a prognostic marker and target for therapy in this aggressive sub-type of DLBCL and TQ may play an important role in the management of DLBCL in the future.

## Introduction

Diffuse large B-cell lymphoma (DLBCL) is the most common type of lymphoma accounting for 30–40% of all lymphomas. The treatment of DLBCL has been revolutionized over the last decade with the addition of Rituximab, an anti-CD20 monoclonal antibody in combination with CHOP [1], however, this disease still remains refractory to treatment in 50% of cases [2]. Gene expression studies performed have been able to identify three distinct groups of DLBCL based on their origin at different stages of differentiation[3]. Of the three groups of DLBCL, activated B cell lymphoma (ABC) tends to have a poor 5 year survival rate of 34%[4], [5] as compared to germinal center B-cell (59%) and primary mediastinal B-cell lymphoma (PMBCL) (64%). The hallmark of ABC subtype of DLBCL is activation of the NFκB survival pathway that allows the malignant cell towards plasma cell differentiation [6]. Activation of NFκB pathway occurs when IκBα, an inhibitor of NFκB is degraded by either proteasomal degradation or ubiquitination allowing NFκB to enter the nucleus and exerts its transcriptional activation on growth factors such as interleukins and pro-survival and anti-apoptotic proteins such as Bcl-2, Bcl-Xl, XIAP and Survivin[7], [8], [9].

Thymoquinone (TQ) is a naturally occurring compound that is extracted from *Nigella sativa* Linn [10]. TQ has been shown to possess anti-inflammatory, anti-oxidant and anti-neoplastic activity [11]. TQ has been shown to possess antitumor activities against a broad spectrum of cancer cells, including colon, ovarian, and myeloblastic leukemia[11], [12], [13]. The exact mode of action of TQ is not known, however, it has been shown that TQ induces its anti-tumor effect via release of reactive oxygen species (ROS)[14]. Recently, it has been shown that TQ enhances the effect of doxorubicin and oxaliplatin in various cancers[15], [16].

In the present study, we first examined the expression of p-IκBα and its association with clinical and pathological parameters in a cohort of ABC-DLBCL clinical samples in a tissue microarray format. We next investigated the role of NFκB pathway on cell survival in ABC cell lines. We also demonstrated the mode of action of TQ for induction of apoptosis and found that TQ inactivated the NFκB pathway via release of ROS. Finally, in this study, we evaluate the combination therapy of TQ and TRAIL in induction of apoptosis in ABC cell lines.

## Materials and Methods

### Ethics Statement

The Institutional Review Board (IRB) and Research Advisory Council (RAC) of KFSHRC approved this study: RAC # 2060008. A waiver of consent was obtained from IRB and ethics committee as this study was performed on archival samples and the samples were analyzed anonymously.

### Patient Samples and Tissue microarray

Two hundred and thirty one cases of *de novo* DLBCL diagnosed at the King Faisal Specialist Hospital and Research Centre (KFSHRC) and reclassified according to the WHO criteria[17] were included in this study. Archival paraffin blocks were obtained and tissue microarrays (TMA) were constructed as described previously[18]. The institutional review board (IRB) of KFSHRC approved the study (Project RAC# 2060008).

### Immunohistochemistry (IHC)

TMA slides were processed and stained manually. The IHC protocol was followed as mentioned before[19]. Primary antibodies used, their dilutions, and cutoff levels are listed in Table S1 **.** Each TMA spot was assigned an intensity score from 0–3(I_0_, I_1–3_) and proportion of tumor staining for that intensity was recorded as 5% increments from a range of 0–100(P_0_, P_1–3_). A final H score (range 0–300) was obtained by adding the sum of scores obtained for each intensity(I) and proportion(P) of area stained (H score  = I_1_×P_1_+I_2_×P_2_+I_3_×P_3_). X-tile plots were constructed for assessment of biomarker and optimization of cut off points based on outcome as described earlier[19], [20].

### Cell lines

The human ABC cell lines HBL-1, OCI-LY3, RIVA and SUDHL2 were a kind gift from Dr Laura Pasqualucci Institute for Cancer Genetics and the Herbert Irving Comprehensive Cancer Center, Columbia University, New York, USA and have been used previously[21], [22]. The cell lines were cultured in IMDM supplemented with 20% (v/v) fetal bovine serum (FBS), 100 U/ml penicillin, 100 U/ml streptomycin at 37°C in a humidified atmosphere containing 5% CO_2_. All the experiments were performed in either RPMI-1640 or IMDM containing 5% serum. All the cell lines were authenticated in house using human identifiler multiplex kit (Life Technologies, New York, NY).

### Reagents and antibodies

Thymoquinone (TQ), N-acetyl cysteine (NAC) and Bax 6A7 monoclonal antibody were purchased from Sigma Chemical Co (St. Louis, MO, USA). zVAD-fmk were purchased from Calbiochem (San Diego, CA, USA). Antibodies against caspases-8, -9, DR5, Bcl-Xl, and cleaved caspase-3 were purchased from Cell Signaling Technologies (Beverly, MA, USA). Cytochrome c, p65, p-IκBα, IκBα, caspase-3, and PARP antibodies were purchased from Santa Cruz Biotechnology, Inc. (Santa Cruz, CA, USA). Survivn, cIAP-1 and cIAP2 antibodies were purchased from R&D. Beta-actin and XIAP antibodies were purchased from Abcam (Cambridge, England). Annexin V was purchased from Molecular Probes (Eugene OR, USA). Apoptotic DNA-ladder kit was obtained from Roche (Penzberg, Germany).

### MTT assays

MTT assay was performed as described earlier [23]. Briefly, 10^4^ cells were incubated in triplicate with indicated doses of TQ for 24 hour at 37°C. Thereafter, 25 µL MTT solution (5 mg/ml in water) was added and after 24 hour incubation at 37°C, 0.1 mL extraction buffer (20% SDS) was added and optical density (OD) at 590 nm was measured.

### Annexin V staining

ABC cell lines were treated with different concentrations of TQ as described in the legends. Cells were harvested and the percentage apoptosis was measured by flow cytometry after staining with flourescein-conjugated annexin-V and propidium iodide (PI) (Molecular probes, Eugene, OR) as described previously[24].

### Cell lysis and Immunoblotting

Cells were treated with TQ as described in the legends and lysed as previously described[25]. Immunoblotting was performed with different antibodies and visualized by an enhanced chemiluminescence (ECL, Amersham, Illinois, USA) method.

### Detection of Bax conformational changes

This assay was performed as described previously[26]. Briefly, following treatment with indicated reagents for various time points, cells were lysed with Chaps lysis buffer and 500 µg of total protein was incubated with 2 µg of anti-Bax 6A7 monoclonal antibody and 25 µl of protein G-beads and incubated at 4°C overnight. Following washes in lysis buffer, samples were separated by SDS-PAGE, transferred and immunoblotted using N20 Bax polyclonal antibody.

### Assay for cytochrome c release

Release of cytochrome c from mitochondria was assayed as described earlier [21]. Following treatment, cells were lysed and mitochondrial and cytosolic extracts were prepared, immunobotted and probed with cytochrome c antibody.

### Measurement of mitochondrial potential using the JC-1 (5, 5′, 6, 6′-teterachloro-1, 1′, 3,3′- tetraethylbenzimidazolylcarbocyanine iodide) assay

1×10^6^ cells were treated with TQ for 24 hours. Cells were washed with PBS and suspended in mitochondrial incubation buffer (Alexis Corporation, Farmingdale, NY, USA) and 10 µM JCI and mitochondrial membrane potential (% of green and red aggregates) was determined by flow cytometry as described previously [21].

### Gene Silencing using SiRNA

DR5 SiRNA, AKT siRNA and Scrambled control siRNA were purchased from Qiagen. p65 siRNA was purchased from Sant Cruz Biotechnology, Inc. (Santa Cruz, CA, USA). For transient expression, cell lines were transfected by using LipofectAMINE 2000 reagent (Invitrogen, Carlsbad, CA). After incubating the cells for 6 hrs, the lipid and SiRNA complex was removed and fresh growth medium was added. Cells were treated 48 hours after transfection for 24 hours and specific protein levels were determined by Western Blot analysis with specific antibodies.

### Preparation of nuclear extracts for NFκB

Nuclear extracts were prepared according as described previously[27]. Briefly, 1×10^7^ cells were washed with cold PBS and suspended in 0.4 mL hypotonic lysis buffer containing protease inhibitors for 30 minutes. The cells were then lysed with 10% Nonidet P-40.

### Measurement of ROS

We used H_2_DCFDA, a cell permeable fluorescent probe for detection for ROS release as described earlier [28]. Briefly, 1×10^6^ exponentially growing cells were loaded with 10 µM H2DCFDA for 45 minutes at 37°C and then treated with TQ for various time periods in presence of 10 mM NAC. Following incubation, the cells were washed with PBS and green fluorescence intensity in the cells was examined by FACS analysis.

### Electrophoretic mobility shift assay for NFκB

The single-stranded 3′-end biotin-labeled probe containing the NFκB consensus site 5′-AGTTGAGGGGACTTTCCCAGGC-3′, and 3′-TCAACTCCCCTGAAAGGGTCCG5′ were purchased from Metabion (martinsried, Germany). The biotinylated oligonucleotides were annealed by denaturing at 90°C for 1 min and cooled to room temperature for 1 hour. The EMSA binding reactions were performed by utilizing a LightShift chemiluminescent EMSA kit (Pierce, Rockford, IL, USA) as described previously[27].

### Luciferase Reporter Assays

ABC cells were transfected with a β-galactosidase and plasmid pNFκB-LUC linked to a minimal E1B promoter-luciferase gene using the Lipofectamine transfection reagent as per manufacturer's recommended procedure (Qiagen, Valencia, CA). Forty-eight hours after transfection, cells were either pre-treated with 10 mM NAC for 3 hours followed by treatment with 10 µM TQ for 24 hours and luciferase activity was measured using manufacturer's protocol (Promega, Madison, WI). The measured luciferase activities were normalized for β-galactosidase activity for each sample.

### Statistical Analysis

The JMP 9.0 (SAS Institute Inc., Cary, NC) software package was used for data analyses. Survival curves were generated using Kaplan-Meier method, with significance evaluated using the Mantel-Cox log-rank test. Risk ratio was calculated using the Cox proportional hazard model in both univariate and multivariate analyses. Cell line data are presented, as mean ± SD. Comparisons between groups were made with the paired Student's t-test. Values of p<0.05 were considered statistically significant.

## Results

### p-IκB expression in DLBCL patients

**Figure 1:**
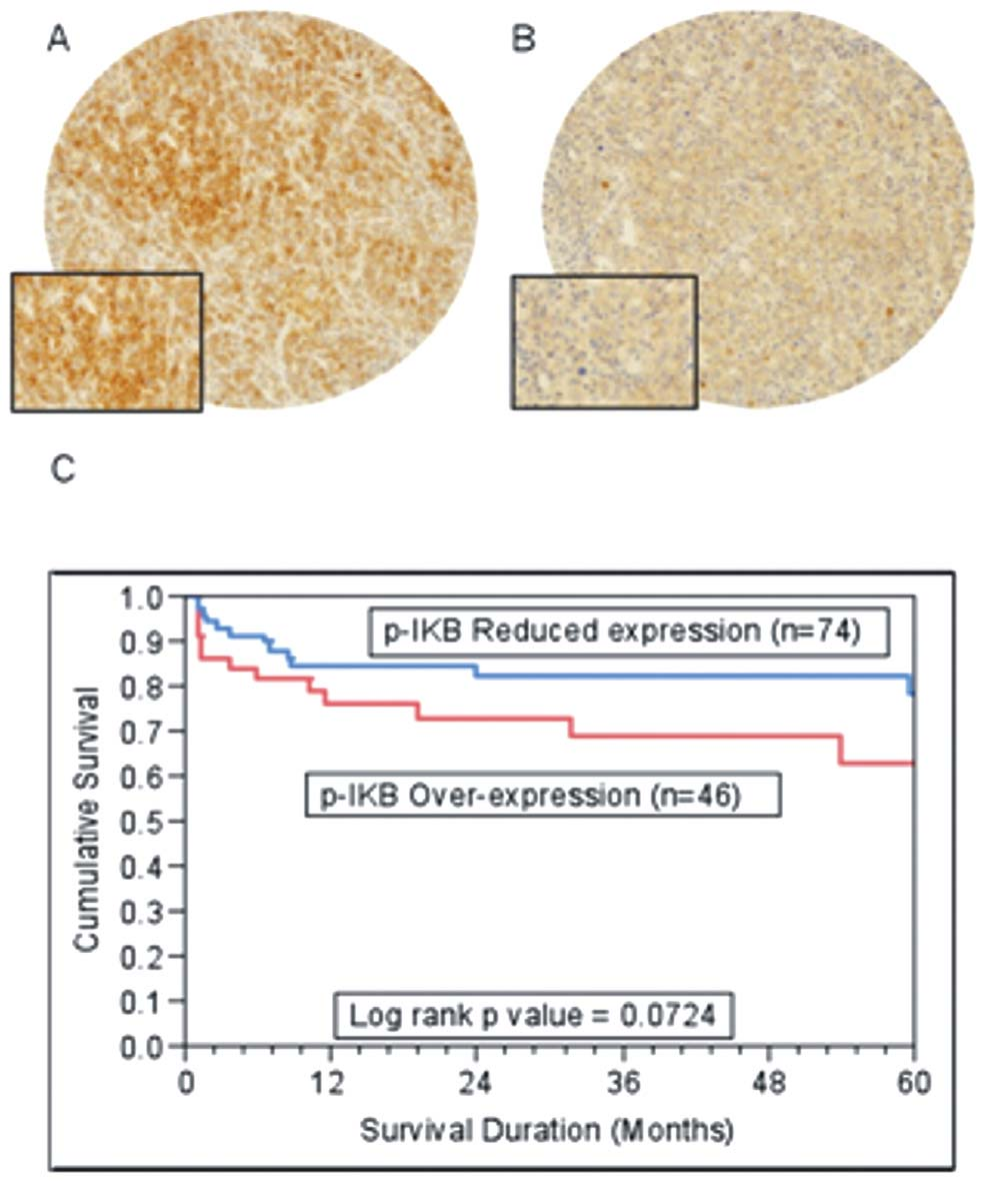
Tissue microarray based immunohistochemical analysis of p-IκB expression in DLBCL patients: DLBCL array spot showing high expression of p-IκB (A) and low expression of p-IκB (B). 20×/0.70 objective on an Olympus BX 51 microscope. (Olympus America Inc, Center Valley, PA, USA. with the inset showing a 40×0.85 aperture magnified view of the same. (C). Impact of p-IκB expression on prognosis in ABC patients. In patients with activated B cell phenotype(ABC) subgroup, p-IκB over expression showed a poor overall survival of 56.3% at 5 years as compared to 78.2% with low p-IκB expression (p = 0.0724).

Activation of NFκB pathway requires prior phosphorylation of IκBα at Ser32 and Ser34 site for it be a target of ubiquitination and degradation releasing p50–p65 heterodimer to translocate to the nucleus [29]. We therefore sought to determine the status of p- IκBα in clinical samples of ABC-DLBCL. Immunohistochemical analysis of p-IκB expression was interpretable in 151 DLBCL spots and p-IκB expression was predominantly seen in the cytoplasmic compartment. The incidence of p-IκB expression in all DLBCL cases and in the ABC subgroup was 38.4% (58/151) and 38.3% (46/120) respectively (Figure 1A). p-IκB expression in the ABC samples did not correlate with age, gender, IPI and Stage (Table 1). Interestingly, p-IκB over expressing ABCs showed a poor outcome (p = 0.0724) of 56.3% as compared to (p = 0.0724) of 78.1% in ABC with reduced p-IκB expression (Figure 1B). Using multivariate analysis in ABC subgroup of DLBCL, for IPI and p-IκB expression, the relative risk was 2.98 for high p-IκB expression (95%; CI 1.17–8.52; p = 0.0213) and 4.08 for high IPI group (95% CI 1.61–11.15; p = 0.0030). Thus, p-IκB over expression was an independent prognostic marker for poor survival in all ABC subgroup of DLBCL (Table S2).

**Table 1:** Correlation of p-IκB expression with clinico-pathological parameters in ABC subtypes of DLBCL samples.

	Total		High		Low		P value
	No.	%	No.	%	No.	%	
No. of Patients^$^	120		46	38.3	74	61.7	
Age (all pts)							
≤50	73	60.8	25	34.2	48	65.8	0.2523
>50	47	39.2	21	44.7	26	55.3	
Sex (all pts)							
Female	47	39.2	20	42.6	27	57.4	0.4464
Male	73	60.8	26	35.6	47	64.4	
Perf. Status (IPI)							
0–2	54	60.7	19	35.2	35	64.8	0.1293
3–4	35	39.3	18	51.4	17	48.6	
Stage (IPI)							
I–II	47	52.8	18	38.3	29	61.7	0.5072
III–IV	42	47.2	19	45.2	23	54.8	
Extranodal (IPI)							
>1 site	41	46.1	12	29.3	29	70.7	0.0283
1 site	48	53.9	25	52.1	23	47.9	
LDH (IPI)							
Normal (<480)	36	40.4	16	44.4	20	55.6	0.6508
High (>480)	53	59.6	21	39.6	32	60.4	
IPI Group@							
Low–Low Inter	58	65.2	24	41.4	34	58.6	0.9596
High Inter–High	31	34.8	13	41.9	18	58.1	
BCL-XL							
Above 230	27	23.5	13	48.1	14	51.9	0.2308
Below = 2300	88	76.5	31	35.2	57	64.8	
XIAP							
Above 150	67	56.3	28	41.8	39	58.2	0.4243
Below = 150	52	43.7	18	34.6	34	65.4	
NFKB (Sc 372)							
Above 0	38	34.6	12	31.6	26	68.4	0.1868
Below = 0	72	65.4	32	44.4	40	55.6	
5-Year Overall Survival				56.3		78.1	0.0724

^@^IPI & Stage information was available only in 89 patients. ?GC vs. ABC Germinal Centre (GC) versus Activated B Cell (ABC) phenotype.^$^Of the 201 Diffuse Large B cell lymphomas, p-IκB results were available in 151 cases and the remaining 50 spots were non informative. Analysis failure for these IHC markers was attributed to missing or non representative spots. Of these 151 TMA spots with available p-IκB data, range of non available spots for rest of the IHC markers ranged from 1 spot for XIAP to 18 spots for XIAP. The remaining cases were considered for correlation analysis.

### TQ inhibited constitutively activated NFκB in ABC cell lines

**Figure 2:**
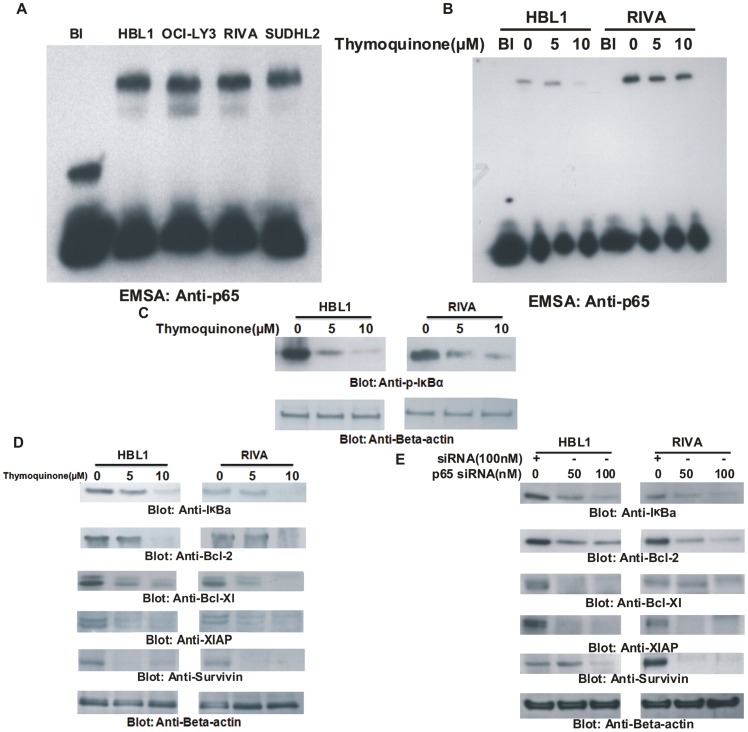
Effect of TQ on constitutive nuclear NFkB in ABC cells. (**A**) **Constitutive expression of NFkB in ABC cells**. Nuclear extracts were prepared and electrophoretic mobility shift assay (EMSA) was performed as described in Materials and Methods. (**B**) **TQ inhibits constitutive nuclear NFkB in ABC cells**. HBL-1 and RIVA cells were treated with indicated doses of TQ for 24 hours. Nuclear extracts were prepared and EMSA was performed to determine the expression of p65 in the nuclear compartment. (**C**) **TQ inactivates IκBα in ABC cell lines**. HBL1 and RIVA cell lines were treated with 5 and 10 µM TQ for 24 hours. Cells were lysed and equal amounts of proteins were separated by SDS-PAGE, transferred to PVDF membrane, and immunoblotted with antibodies against p-IκBα and beta-actin. (**D**) **TQ causes down-regulation of expression of down-stream targets of p65**. HBL-1 and RIVA cells were treated with 5 and 10 µM TQ for 24 hours. Cells were lysed and equal amounts of proteins were separated by SDS-PAGE, transferred to PVDF membrane, and immunoblotted with antibodies against IκBα, Bcl-2, Bcl-Xl, XIAP, Survivin and actin as indicated. (**E**) **Transcriptional down-regulation of p65 causes decreased expression of p65 targets in ABC cells.** HBL-1 and RIVA cells were transfected with siRNA against p65 for 48 hours. Following transfection, cells were lysed and equal amounts of proteins were separated by SDS-PAGE, transferred to PVDF membrane, and immunoblotted with antibodies against IκBα, Bcl-2, Bcl-Xl, XIAP, Survivin and Beta-actin.

As NFκB survival pathway has been found to be constitutively activated in cancers cell lines[27], we were interested in investigating whether there was constitutive activation of NFκB in ABC cell lines. As shown in Figure 2A, constitutively activated NFκB was detected in all ABC cell lines as assessed by EMSA. Next, we sought to determine whether TQ treatment suppressed constitutive activation of NFκB in ABC cell lines. HBL-1 and RIVA cell line were treated with 5 and 10 µM TQ for 24 hours and nuclear extracts were analyzed with EMSA. TQ treatment decreased translocation of p65 in nuclear compartment in a dose dependent manner in both the cell lines (Figure 2B). We also examined the phosphorylation status of p65 in the nuclear compartment following treatment with TQ and found constitutive activation of p65 in ABC cells and TQ treatment inactivated phosphorylation of p65 in a dose dependent manner (Figure S1). We next investigated whether TQ treatment inactivated IκBα phosphorylation. As shown in Figure 2C, untreated samples demonstrated constitutive activation of Ser32-phosphorylated IκBα and treatment with TQ de-phosphorylated IκBα. P65 subunit of NFκB has been shown to transcriptionally up-regulate the expression of various anti-apoptotic and pro-survival gene[30]. We were therefore interested to determine whether TQ treatment could down-regulated expression of down-stream targets of p65. As shown in Figure 2D, TQ treatment down-regulated the expression of IκBα, Bcl-2, Bcl-Xl, XIAP and Survivin in a dose dependent manner in both the cell lines tested. This data was further confirmed by targeting ABC cell lines with siRNA against p65. As shown in Figure 2E, similar response was observed confirming the specificity of TQ in the treatment of ABC.

### TQ causes Reactive Oxygen Species (ROS) release in ABC cells

**Figure 3:**
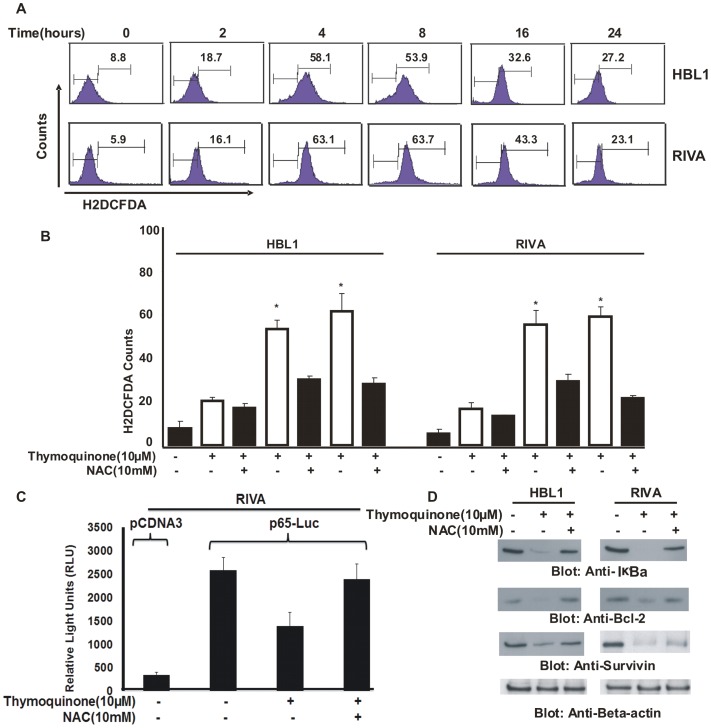
Thymoquinone-induced generation of ROS in ABC cells. (**A**) **Thymoquinone increases ROS generation in ABC cells.** HBL-1 and RIVA cells were incubated in the absence or presence of 10 µM TQ for indicated time periods. After washing with PBS, cells were incubated with 10 µM H2DCFDA and incubated in the dark for 30 minutes at 37°C as described in material and method. Cells were washed in PBS, re-suspended in PBS and analyzed by flow cytometry. (**B**) **NAC prevents TQ-induced ROS release in ABC cells.** HBL-1 and RIVA cell lines were pre-treated with 10 mM NAC for 21hours followed by treatment with 10 µM TQ for various time periods. Cells were washed in PBS and incubated with 10 µM H2DCFDA and incubated in the dark for 30 minutes at 37°C. Cells were washed in PBS, re-suspended in PBS and analyzed by flow cytometry. Bar graph displays the mean +/− SD (standard deviation) of three independent experiments, * p<0.05, statistically significant (Students *t*-test). (**C**) (**C**) **NFκB activity is down-regulated by TQ as measured by Luciferase assay.** RIVA cells were transiently transfected with p65-LUC plasmid for 48 hours. After transfection, RIVA cells were divided into three groups; one group was left untreated as control, the second group was treated with 10 µM TQ for 24 hours and third group was pretreated with 10 mM NAC for 3 hours followed by treatment with 10 µM TQ for 24 hours. After 24 hours, cells were lysed in luciferase lysis buffer and luciferase activity was measured. pCDNA plasmid was used as transfection control and β-galactosidase was used to normalize the transfection efficiency. **(D)** **NAC prevents TQ-induced down-regulation of targets of p65.** HBL-1 and RIVA cells were pre-treated with 10 mM NAC for three hours followed by treatment with 10 µM TQ for 24 hours. Cells were lysed and proteins were immunoblotted with antibodies against IκBα, Bcl-2 and Survivin.

TQ has been previously shown to cause release of ROS in cancer [13], [14]. To investigate this, we treated ABC cell lines with 10 µM TQ for various time periods and cells were stained with H2DCFDA, a detector of ROS release and analyzed by flow cytometry. Both HBL-1 and RIVA cell line demonstrated release of ROS starting as early as 2 hours, peaking between 4 and 8 hours and slowly tapering up to 24 hours (Figure 3A). To confirm ROS release, we pre-treated HBL-1 and RIVA cells with N-acetyl cysteine (NAC), a scavenger of ROS release for 3 hours and then treated the cells with 10 µM TQ for up to 8 hours. As shown in Figure 3B, ROS release was blocked in cells pre-treated with NAC. We next examined whether TQ treatment can repress constitutive active NFκB gene expression in reporter assays. RIVA cells were transiently transfected with NFκB reporter construct for 48 hours and then treatment with TQ for 24 hours and luciferase activity was determined. As shown in Figure 3C, constitutive NFκB luciferase activity was seen in untreated cells while TQ treatment abrogated the constitutive NFκB-reporter activity. Interestingly, NAC pre-treatment of RIVA prevented TQ-induced abrogation of luciferase activity. Finally, to confirm that TQ-induced inactivation of NFκB is related to ROS release, we pre-treated HBL-1 and RIVA cells with NAC for three hours and then treated with 10 µM TQ for 24 hours. As shown in Figure 3D, NAC pre-treatment blocked TQ-induced down-regulation of NFκB targets; IκBα, Bcl-2 and Survivin confirming the mode of action of TQ being ROS release in ABC cells. Finally, to determine the mode of generation of ROS by TQ, we pre-treated ABC cell lines with either catalase, superoxide dismutase and NAC for three hours followed by treatment with 10 µM TQ for 24 hours and examined the cells for apoptosis by flow cytometry. As shown in Figure S2, catalase and SOD partially blocked TQ-induced apoptosis, however when both the inhibitors were used in combination, there was efficient inhibition of apoptosis. These data suggest that TQ generates superoxide anion (O_2_-) and hydrogen peroxide (H_2_O_2_) radicals.

### TQ-induced activation of mitochondrial apoptotic pathway in ABC cells

**Figure 4:**
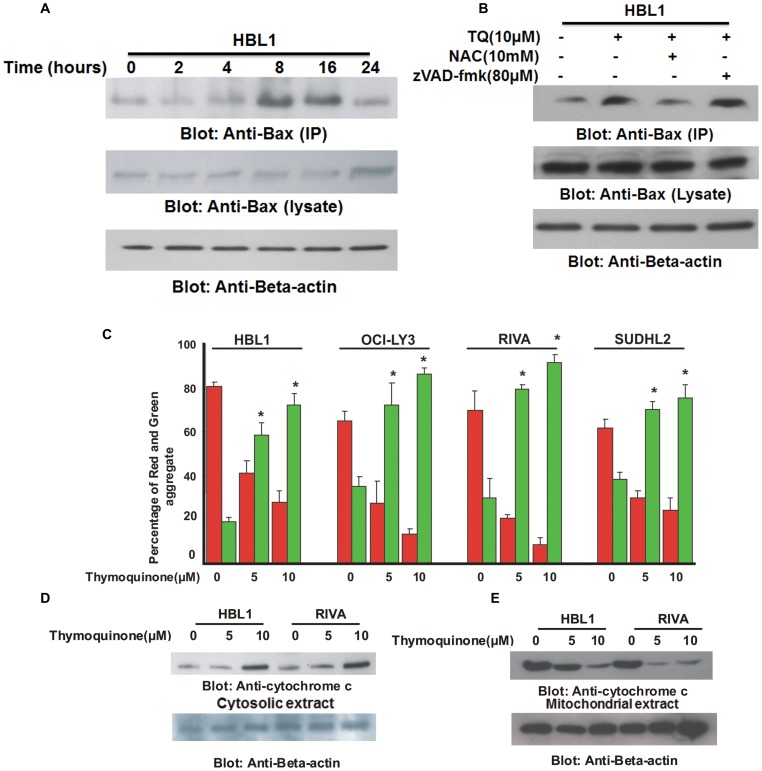
TQ-induced mitochondrial signaling pathway in ABC cells. (A) TQ treatment causes Bax conformational changes in ABC cells. After treating with 10 µM TQ for indicated time periods, HBL-1 cells were lysed and immuno-precipitated with anti-Bax 6A7 antibody for detection of conformationally changed Bax protein. In addition, the total cell lysates were immuno-blotted with specific anti-Bax polyclonal antibody. (B) NAC prevents TQ-induced Bax conformational changes in ABC cells. HBL-1 cells were pre-treated with either, 10 mM NAC and 80 µM z-VAD/fmk for 3 hours and subsequently treated with 10 µM TQ for 8 hours. Cells were lysed and immunoprecipitated with anti-Bax 6A7 antibody and proteins were immunoblotted with Bax rabbit polyclonal antibody. (C) TQ treatment causes change in mitochondrial membrane potential in ABC cells. ABC cells were treated with and without 5 and 10 µM TQ for 24 hours. Live cells with intact mitochondrial membrane potential and dead cells with lost mitochondrial membrane potential was measured by JC-1 staining and analyzed by flow cytometry as described in Materials and Methods. Bar graph displays the mean +/− SD (standard deviation) of three independent experiments, * p<0.05, statistically significant (Students *t*-test). (D) TQ treatment causes release of cytochrome c from mitochondria into cytosole. HBL-1 and RIVA cells were treated with 5 and 10 µM TQ for 24 hours. Mitochondrial free cytosolic fractions were isolated and immunoblotted with antibody against cytochrome c and Beta-actin.

The anti-apoptotic members of the Bcl-2 family, Bcl-2 and Bcl-Xl are transcriptional targets of NFκB and TQ treatment of ABC cells led to down-regulation of these proteins (Figure 2D). This leads to conformational changes in the pro-apoptotic protein; Bax. To confirm this, we treated HBL-1 cell line with 10 µM TQ for various time periods and lysed with 1.0% Chaps lysis buffer; lysates were immuno-precipitated with BAX 6A7 antibody that recognizes only the conformationally changed Bax protein. The detergent Chaps has been shown to retain Bax in its native conformation [31]. As shown in Figure 4A, expression of conformationally changed Bax increased in 8 and 16 hours after treatment with 10 µM TQ. To assess whether conformational changes in Bax protein is due to TQ-induced ROS release or activation of caspases, we pre-treated HBL-1 cells with either 10 mM NAC or 80 µM zVAD-fmk, an universal inhibitor of caspases for 3 hours followed by treatment with 10 µM TQ for 8 hours. NAC pre-treatment blocked TQ-induced conformational changes of Bax protein while zVAD-fmk pre-treatment did not alter changes in Bax protein (Figure 4B). This data clearly suggested that ROS release secondary to TQ treatment induced Bax conformational changes in ABC cell lines.

Once Bax is conformationally changed, it translocates to the mitochondrial membrane and causes changes in the mitochondrial membrane potential leading to release of cytochrome c into cytosole. We therefore treated ABC cell lines with 5 and 10 µM TQ for 24 hours and stained the cells with JC1 dye and analyzed the cells by flow cytometry. As shown in Figure 4C, TQ treatment resulted in loss of mitochondrial membrane potential as measured by JC1 stained green florescence depicting apoptotic cells. Additionally, changes in mitochondrial membrane potential led to release of cytochrome c into cytosole from the mitochondria suggesting that the mitochondrial pathway was being activated following TQ treatment (Figure 4D and E).

**Figure 5:**
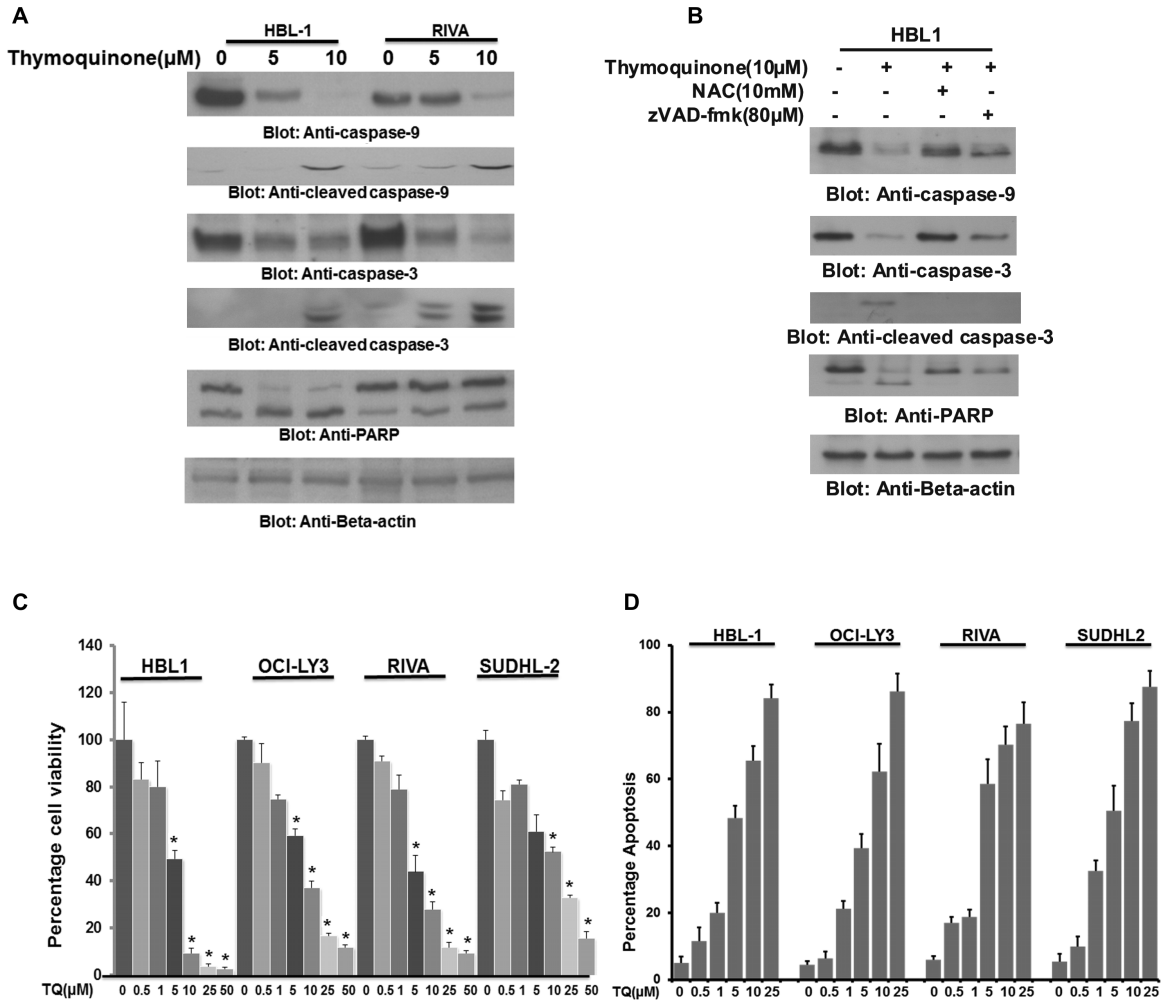
TQ-induced caspases dependent apoptosis in ABC cells. (A) TQ treatment causes activation and cleavage of caspases in ABC cells. HBL-1 and RIVA cells were treated with and without 5 and 10 µM TQ for 24 hours. Cells were lysed and equal amounts of proteins were immunoblotted with antibodies against caspase-9, caspase-3, PARP and Beta-actin. (B) TQ-induced caspases activation and cleavage is blocked by NAC and caspases inhibitor. HBL-1 cells were pretreated with either 10 mM NAC or 80 µM z-VAD for 3 hours and subsequently treated with 10 µM TQ for 24 hours. Cells were lysed and equal amounts of proteins were immunoblotted with antibodies against caspase-9, caspase-3 cleaved caspase-3, PARP and beta-actin. (C) TQ suppresses growth of ABC cells. ABC cell lines were incubated with 0–50 µM TQ for 24 hours. Cell viability was measured by MTT assays as described in Materials and Methods. The graph displays the mean +/− SD (standard deviation) of three independent experiments, * p<0.05, statistically significant (Students *t*-test). (D) TQ treatment induces apoptosis in ABC cell lines. ABC cells were treated with 5 and 10 µM TQ (as indicated) for 24 hours and cells were subsequently stained with flourescein-conjugated annexin-V and propidium iodide (PI) and analyzed by flow cytometry.

Activation of the mitochondrial apoptotic pathway leads to activation and cleavage of caspases-9 and -3 in response to various apoptotic-inducing agents [21]. Treatment of ABC cell lines with TQ also activated and cleaved caspases-9 and -3 and PARP in a dose dependent manner (Figure 5A). In order to determine whether activation and cleavage of caspases is dependent on ROS release, we pre-treated HBL-1 cell line with either 10 mM NAC or 80 µM zVAD-fmk for 3 hours followed by treatment with TQ for 24 hours. As shown in Figure 5B, both NAC as well as zVAD-fmk pre-treatment were able to block TQ-induced activation of caspases-9, -3 and cleavage of PARP.

We also wanted to determine whether TQ treatment could inhibit cell viability and induce apoptosis in ABC cell lines. We treated ABC cell lines with incremental doses of TQ for 24 hours and analyzed the cells for cell viability by MTT assay. As shown in Figure 5C, there was a dose dependent inhibition of cell viability in all the ABC cell lines tested following treatment with TQ. We were also able to detect dose dependent apoptosis in ABC cell lines treated with increasing doses of TQ for 24 hours (Figure 5D). These doses have been previously shown to be non-toxic to peripheral blood mononuclear cells isolated from normal healthy individuals[14]. These data clearly suggested that TQ activated the mitochondrial apoptotic pathway to induce apoptosis in ABC cells.

### TQ-induced ROS generation causes up-regulation of Death Receptor 5 (DR5)

**Figure 6:**
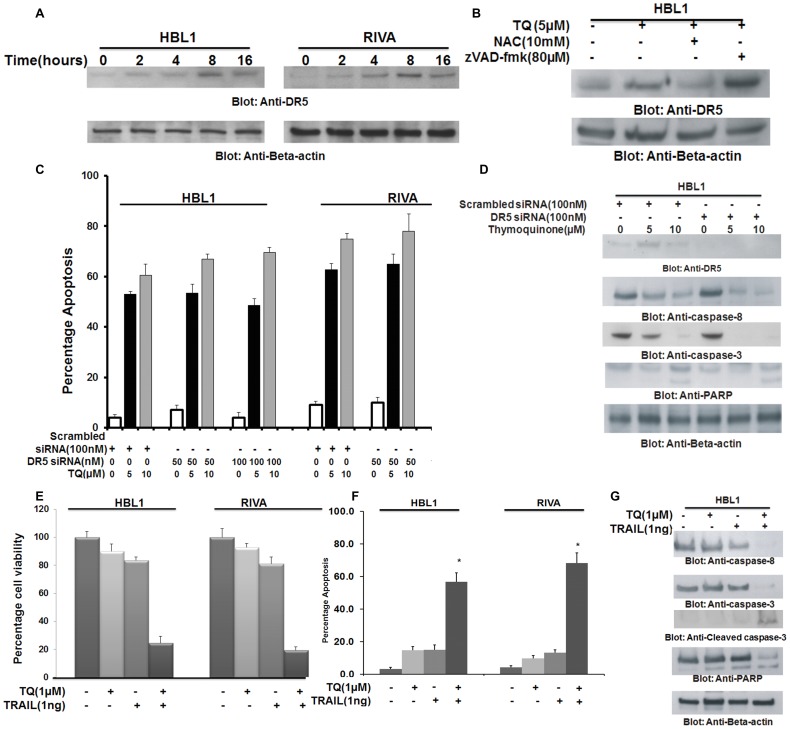
TQ-induced up-regulation of DR5 does not play a part in TQ-induced apoptosis. (A) TQ treatment causes up-regulation of DR5 in ABC cells. HBL1 and RIVA cells were treated with 10 µM TQ for indicated time periods. After cell lysis, equal amounts of proteins were immuno-blotted with antibodies against DR5 and beta actin. (B) TQ-induced DR5 up-regulation is ROS dependent. HBL-1 cells were pre-treated with either 10 mM NAC or 80 µM z-VAD for 3 hours and subsequently treated with 10 µM TQ for 24 hours. Cells were lysed and equal amounts of proteins were immunoblotted with antibodies against DR5 and beta-actin. (C and D) TQ-induced apoptosis is not DR5 dependent. (C) HBL-1 and RIVA cells were either transfected with 50 and 100 nM siRNA, specific against DR5 or scrambled siRNA for 48 hours. Cells were then treated with 5 and 10 µM TQ for 24 hours, following which cells were stained with fluorescent-conjugated Annexin V/PI and analyzed by flow cytometry. Bar graph displays the mean +/− SD (standard deviation) of three independent experiments, * p<0.05, statistically significant (Students *t*-test). (D) HBL-1 and RIVA cells were either transfected with 50 and 100 nM siRNA, specific against DR5 or scrambled siRNA for 48 hours and treated with 5 and 10 µM TQ for 24 hours. Cells were lysed and equal amounts of proteins were immuno-blotted with antibodies against DR5, caspase-8, caspase-3, PARP and beta-actin. (E): Combination treatment with sub-optimal doses of TQ and TRAIL induce synergistic inhibition of cell viability and apoptosis in ABC cells. HBL-1 and RIVA cells were treated with either 1 µM TQ in the presence and absence of 1 ng TRAIL for 24 hours. Following treatment, cells were analyzed for cell viability by MTT assay as described in material and methods. Bar graph displays the mean +/− SD (standard deviation) of three independent experiments, * p<0.05, statistically significant (Students *t*-test). (F) HBL-1 and RIVA cells were treated with either 1 µM TQ in the presence and absence of 1 ng TRAIL for 24 hours. Following treatment, cells were stained with fluorescent-conjugated Annexin V/PI and analyzed by flow cytometry (G) HBL-1 cells were treated with either 1 µM TQ in the presence and absence of 1 ng TRAIL for 24 hours. Following treatment, cells were lysed and equal amounts of proteins were immuno-blotted with antibodies against caspase-8, caspase-3, PARP, and beta-actin.

Recent studies have shown that DR5 expression is up-regulated by ROS generated by TQ[14]. In view of these findings, we sought to determine whether TQ-generated free radicals modulate the expression of DR5 in ABC cells. We first treated HBL-1 and RIVA cell lines with 5 µM TQ for indicated time periods and expression of DR5 was determined by immune-blotting. DR5 was found to be up-regulated within 8 hours of TQ treatment in both the cell lines (Figure 6A). We next sought to determine whether ROS release secondary to TQ treatment led to up-regulation of DR5 or were it caspases dependent. HBL-1 cell line was either treated with 10 mM NAC or 80 µM zVAD-fmk for 3 hours followed by treatment with 10 µM TQ for 24 hours. As shown in Figure 6B, NAC pre-treatment was able to block TQ-induced up-regulation of DR5 while caspases inhibitor failed to block DR5 up-regulation. This data clearly indicated that DR5 up-regulation is dependent on TQ-induced ROS release in ABC cells.

### DR5 up-regulation does not contribute to TQ-induced apoptosis in ABC cells

DR5, one of the receptors of TRAIL is required for activation of the extrinsic apoptotic pathway [32], however, it has been previously been shown that DR5 does not a play a role in TQ-induced apoptosis [14]. To confirm this, we transiently transfected HBL-1 and RIVA cells with either scrambled or DR5 targeting siRNA followed by treatment with TQ for 24 hours. Knock-down of DR5 expression did not affect the apoptotic response of ABC cells to TQ treatment as assessed by annexin v/PI dual staining (Figure 6C) nor did it affect the activation and cleavage of caspases-8, -3 and PARP (Figure 6D) suggesting that DR5 does not play a role in TQ-induced apoptosis in ABC cells.

### Synergistic activity of TRAIL and TQ to induce apoptosis in ABC cell lines

Even though DR5 did not play an active role in TQ-induced apoptosis in ABC cells, it presented as an additional attractive target for the treatment of ABC cells. We therefore sought to determine whether co-treatment of TQ and TRAIL, a death ligand, at sub-optimal doses could induce a more potent apoptosis in ABC cells. As shown in Table S3 and Figure S3, using Chou and Talalay method [14], [33], we found that 1 µM TQ and 1 ng super killer TRAIL exerted the maximum synergistic apoptotic response in HBL-1 cells (combination index 0.359) and RIVA cells (combination index 0.168) both the values being less than 0.5 suggesting a strong synergistic response[33]. We therefore co-treated DLBCL cells with 1 µM TQ and 1 nM TRAIL and assessed cell viability by MTT assay and apoptosis. As shown in Figure 6E, neither TQ nor TRAIL treatment inhibited cell viability alone, however, when both the drugs were given in combination, there was sufficient inhibition of cell viability and induction of apoptosis (Figure 6F) along with activation and cleavage of caspases-8, -3 and PARP in ABC cell (Figure 6G). These data clearly indicate that combination of TQ and TRAIL efficiently induces apoptosis in ABC cells.

## Discussion

Although DLBCL can be morphologically similar, gene expression profiling has demonstrated molecularly distinct subtypes with different clinical behavior and outcome[34]. One of the main factors that is associated with poor prognosis of ABC sub-type of DLBCL is the constitutive activation of NFκB pathway due to its ability to block apoptotic response to different chemotherapeutic agents [35]. Understanding the aetiology of NF-κB activation can provide a rationale for targeting NF-κB in the ABC subtype of DLBCL that is characterized by constitutive NF-κB activation and shows resistance to current therapeutic modalities. In our earlier study, we had shown nuclear factor kappa B expression was seen in 25% of DLBCL, was associated with activated B cell phenotype and poor survival[36]. In the current study, we found that p-IkB expression is also an independent prognostic marker for poor survival in ABC subgroup of DLBCL. Considering the potential prognostic and therapeutic role of NF-kB, p-IkB can be a promising marker along with NF-kB in a panel of molecular markers to detect DLBCL with NF-kB activation.

Our *in-vitro* studies have shown that panel of ABC cell lines derived also exhibit constitutive NFκB activity. In this study, we have investigated the molecular mechanism of NFκB mediated anti-apoptotic role in ABC cell lines using Thymoquinone (TQ), an active constituent of black seed that has well-documented pro-apoptotic properties in a variety of cell types, including cells of hematopoietic origin [12], [13], [37], [38]. Our study indicates that TQ treatment inhibits constitutive activation of NFκB suggesting an important role in inhibiting the growth and viability of ABC *in vitro*. By inhibiting the constitutive activity of NFκB, the anti-apoptotic transcriptional targets of NFκB also get down-regulated thereby allowing the pro-apoptotic proteins to assert its apoptotic effects. The anti-apoptotic targets of NFκB, Bcl-2 and Bcl-Xl belong to Bcl-2 family of proteins and have been shown to exert their anti-apoptotic effect acting as guardians of mitochondria by in-activating Bax and Bak, the pro-apoptotic members of the Bcl-2 family [39]. On the other hand, XIAP inhibits apoptosis via interacting with caspases-9 and -3 thereby inhibiting their activation [40]. By down-regulating the NFκB activity, TQ treatment also down-regulates the expression of Bcl-2, Bcl-Xl and XIAP leading to mitochondrial-induced apoptosis via activation and cleavage of caspases in ABC cells.

There are studies that have shown that ROS release sensitizes cancer cells to TRAIL induced apoptosis via up-regulation of DR5 in various cancers [41], [42], [43], [44], [45]. We also show that treatment of ABC cells with TQ causes up-regulation of DR5. Even though, we find up-regulation of DR5, DR5 alone does not play a role in TQ-induced apoptosis in ABC cells because, even after knocking down the expression of DR5 in ABC cells, there was efficient apoptosis following TQ treatment. However, DR5 up-regulation following TQ treatment does offer an attractive therapeutic target for the treatment of ABC cells. We have previously shown that TQ-induced up-regulation can be targeted with TRAIL to induce a synergistic apoptotic response in primary effusion lymphoma cells[14]. In concordance, combination of TQ and TRAIL at sub-optimal doses induced significant apoptosis as compared to treatment as a single agent in ABC cells. The benefit of targeting ABC cells with a combination of TQ and TRAIL is that both the intrinsic as well as the extrinsic apoptotic pathways can be activated simultaneously to elicit a more potent apoptotic response. Another reason of using this combination at sub-optimal doses is that TRAIL at high concentrations has been found to be toxic to normal cells of the body [46], however, at sub-optimal doses, TRAIL does not elicit cytotoxicity towards normal cells thereby making it an attractive drug to be used in conjunction with other inhibitors or chemotherapeutic agents.

Treatment of ABC subtype of DLBCL still remains a major challenge despite improvement in therapy including addition of Rituximab with CHOP. In this manuscript, we have highlighted a different mechanism of targeting these aggressive lymphomas. Targeting of constitutively active survival pathways such as NFκB with agents such as TQ in cancer allow selective killing of these aggressive cells with minimal targeting of their normal counterparts thereby decreasing the chances of toxicity. Therefore the cytotoxic inducing ability of TQ in cancer could make it a potentially effective chemo-preventive and/or therapeutic agent for the treatment of these lymphomas. In addition, combination treatment with TQ and TRAIL allows further reduction in the dosage of these agents thereby improving the response and decreasing the toxicity. Based on our data, further studies in this direction are warranted.

## Supporting Information

Figure S1 **TQ inactivated p65 in the nuclear compartment in ABC cells.** HBL1 and RIVA cells were treated with 5 and 10 µM TQ for 24 hours. Following treatment, cells were nuclear extracts were prepared and immunoblotted with antibodies against p-p65 and Alpha-Tubulin for equal loading.
(TIF)

Figure S2 **TQ-induced apoptosis is blocked by combination of catalase and superoxide dismutase (SOD) in ABC cells.** HBL1 and RIVA cells were pre-treated with 100 units/ml catalase, 100 units/ml SOD, combination of catalase and SOD and NAC for three hours followed by treatment with 10 µM TQ for 24 hours. Following treatment, cells were stained with fluorescein-conjugated annexin v/PI and analyzed for apoptosis by flow cytometry. Bar graph displays the mean +/− SD (standard deviation) of three independent experiments.
(TIF)

Figure S3 **Synergistic apoptotic response of TQ and TRAIL in ABC cells.** (A and B) HBL-1 and RIVA cells were treated with various combinations of TQ and TRAIL alone or in combination for 24 hours and dose effect was measured using calcusyn software. (C and D) Five different concentrations of TQ (0.5 to 25 µM) and TRAIL (0.5 to 25 ng) were used together in different combinations as shown in the Figure C and D (1–5) to determine the Fractional effect of combination treatment with TQ and TRAIL and graphs were generated using Calcusyn software. Apoptotic response were analyzed as mean ± SD values normalized to control. Combination indices were calculated using Chou and Talalay methodology.
(TIF)

Table S1 **Antibodies used for tissue micro array Immunohistochemical analysis.** List of antibodies, clones, dilution, antigen retrieval and detection method used for immunohistochemistry are indicated in Table S1.
(DOCX)

Table S2 **Cox regression analysis for overall survival of patients with diffuse large B-cell lymphoma –p-IKBα in ABC Group.** Univariate and Multivariate analysis were performed to determine the relative risk and confirm the utility of p-IKBα as an independent prognostic marker.
(DOCX)

Table S3 **Combination index calculation using Chou and Talalay method in ABC cell lines.** HBL1 and RIVA cell lines were treated with various doses of TQ and TRAIL alone or in combination and Fraction effect (Fa), Combination Index (CI) and Dose Reduction Index (DRI) are indicated in Table S3.
(DOCX)
