# Supplementary figures and images for: Phosphorylated IκBα Predicts Poor Prognosis in Activated B-Cell Lymphoma and Its Inhibition with Thymoquinone Induces Apoptosis via ROS Release
Source: PLoS One. 2013 Mar 28;8(3):e60540. doi: 10.1371/journal.pone.0060540 (PMC3610815; doi:10.1371/journal.pone.0060540)

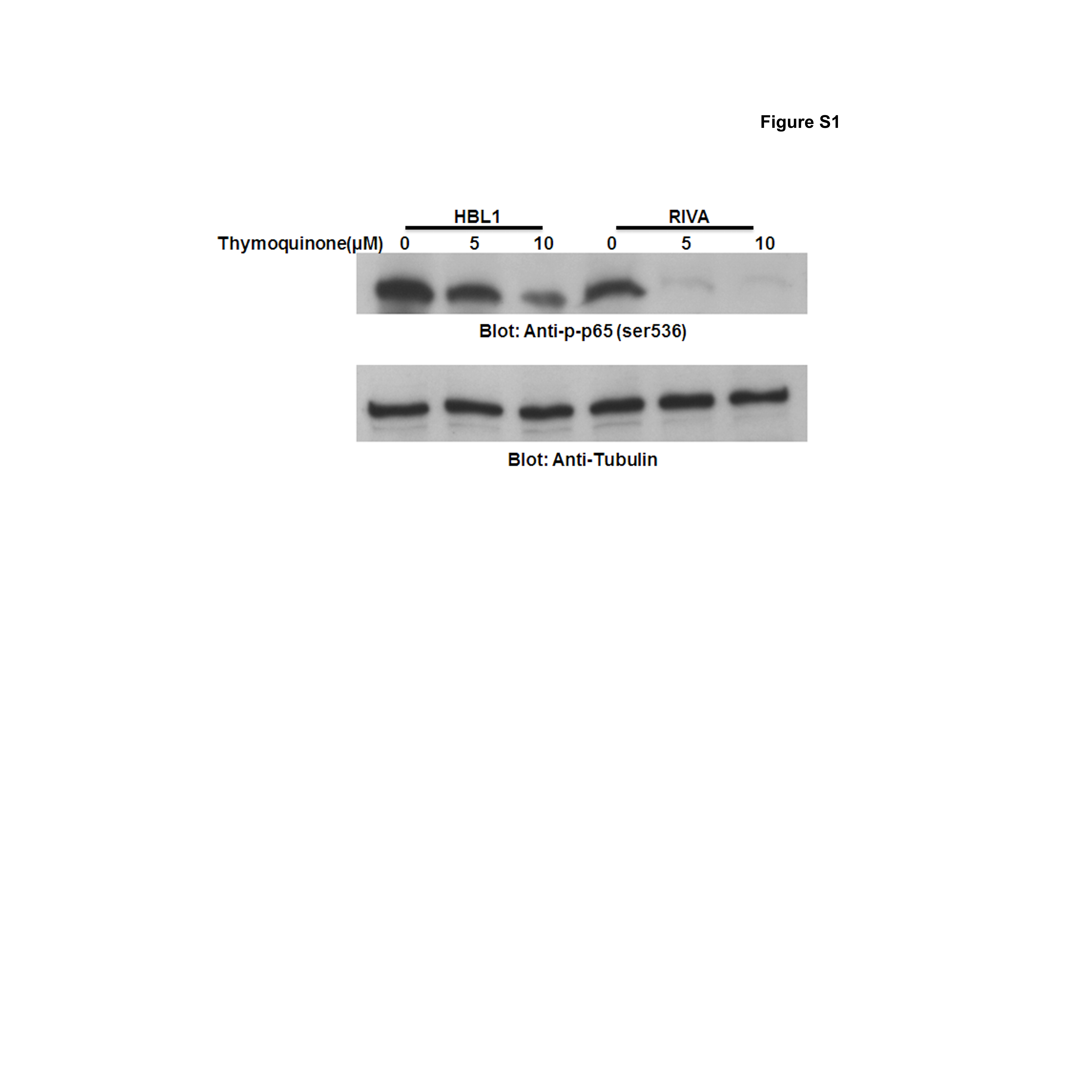

Supplement: Figure S1 — TQ inactivated p65 in the nuclear compartment in ABC cells. HBL1 and RIVA cells were treated with 5 and 10 µM TQ for 24 hours. Following treatment, cells were nuclear extracts were prepared and immunoblotted with antibodies against p-p65 and Alpha-Tubulin for equal loading. (TIF) [file pone.0060540.s001.tif]

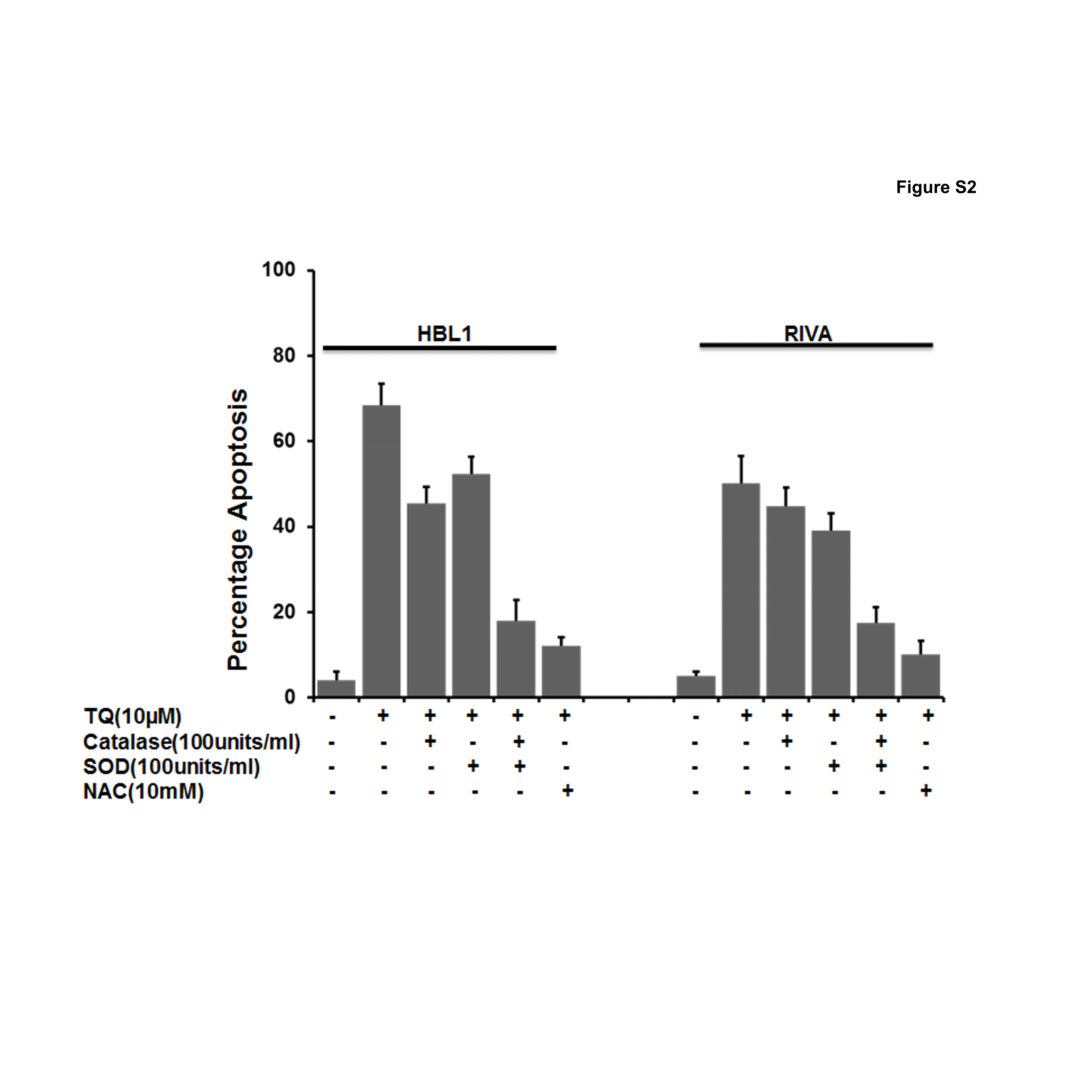

Supplement: Figure S2 — TQ-induced apoptosis is blocked by combination of catalase and superoxide dismutase (SOD) in ABC cells. HBL1 and RIVA cells were pre-treated with 100 units/ml catalase, 100 units/ml SOD, combination of catalase and SOD and NAC for three hours followed by treatment with 10 µM TQ for 24 hours. Following treatment, cells were stained with fluorescein-conjugated annexin v/PI and analyzed for apoptosis by flow cytometry. Bar graph displays the mean +/− SD (standard deviation) of three independent experiments. (TIF) [file pone.0060540.s002.tif]

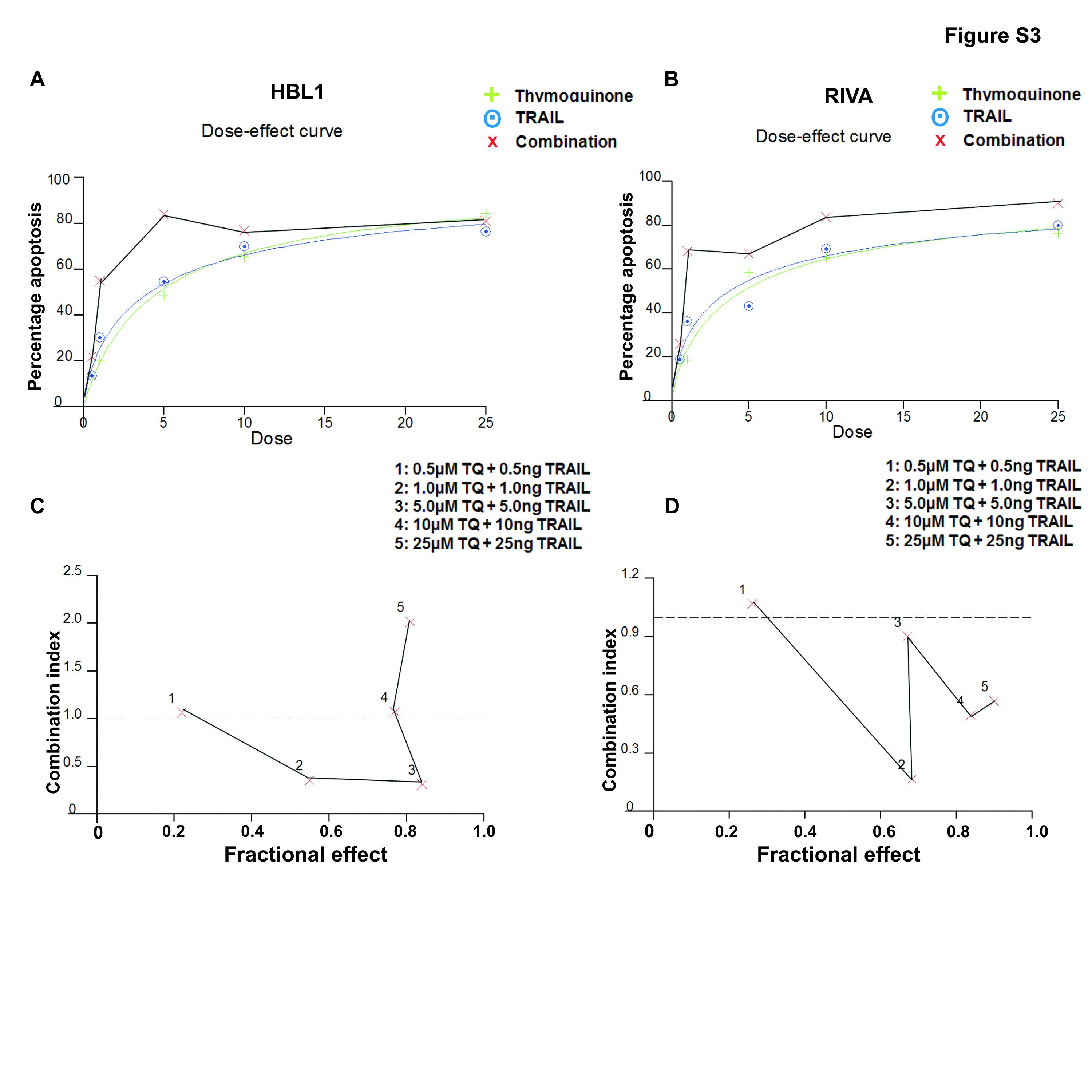

Supplement: Figure S3 — Synergistic apoptotic response of TQ and TRAIL in ABC cells. (A and B) HBL-1 and RIVA cells were treated with various combinations of TQ and TRAIL alone or in combination for 24 hours and dose effect was measured using calcusyn software. (C and D) Five different concentrations of TQ (0.5 to 25 µM) and TRAIL (0.5 to 25 ng) were used together in different combinations as shown in the Figure C and D (1–5) to determine the Fractional effect of combination treatment with TQ and TRAIL and graphs were generated using Calcusyn software. Apoptotic response were analyzed as mean ± SD values normalized to control. Combination indices were calculated using Chou and Talalay methodology. (TIF) [file pone.0060540.s003.tif]
