# Supplementary material for: Phosphorylated IκBα Predicts Poor Prognosis in Activated B-Cell Lymphoma and Its Inhibition with Thymoquinone Induces Apoptosis via ROS Release
Source: PLoS One. 2013 Mar 28;8(3):e60540. doi: 10.1371/journal.pone.0060540 (PMC3610815; doi:10.1371/journal.pone.0060540)
Supplement: Table S1 — Antibodies used for tissue micro array Immunohistochemical analysis. List of antibodies, clones, dilution, antigen retrieval and detection method used for immunohistochemistry are indicated in Table S1. (DOCX) [file pone.0060540.s004.docx]

Table S1**. Antibodies used for tissue micro array Immunohistochemical analysis.**

| **Antibody**  **(Subcellular Localization)** | **Clone** | **Company** | **Source** | **Dilution*** | **Antigen Retrieval** | **Detection System** |
| --- | --- | --- | --- | --- | --- | --- |
| p-IкB | Polyclonal | CST | Rabbit | 1:100 | pH9,  ^PC | Envision+ |
| BCLXL | 54H6 | CST | Rabbit | 1:800 | pH9,  ^PC | Envision+ |
| XIAP | 48 | BD | Mouse | 1:300 | pH9,  ^PC | Envision+ |
| NFкB | Polyclonal | SCBT | Rabbit | 1:500 | pH6,  ^PC | Envision+ |

*O/N-Over night incubation; ^ PC-Pressure cooker; @MW-Microwave
