# Supplementary material for: Phosphorylated IκBα Predicts Poor Prognosis in Activated B-Cell Lymphoma and Its Inhibition with Thymoquinone Induces Apoptosis via ROS Release
Source: PLoS One. 2013 Mar 28;8(3):e60540. doi: 10.1371/journal.pone.0060540 (PMC3610815; doi:10.1371/journal.pone.0060540)
Supplement: Table S2 — Cox regression analysis for overall survival of patients with diffuse large B-cell lymphoma –p-IKBα in ABC Group. Univariate and Multivariate analysis were performed to determine the relative risk and confirm the utility of p-IKBα as an independent prognostic marker. (DOCX) [file pone.0060540.s005.docx]

| Table S2. **Cox regression analysis for overall survival of patients with diffuse large B-cell lymphoma –p-PIKB Alpha in ABC Group.** | | | | | | |
| --- | --- | --- | --- | --- | --- | --- |
| *Univariate Analysis* | | | | *Multivariate Analysis* | | |
| Relative Risk 95% CI p value | | | | Relative risk 95%CI p value | | |
| International Prognostic Index | 7.10 | 3.04-18.53 | <0.0001 | 4.08 | 1.61-11.15 | 0.0030 |
| High p-IκBα | 1.95 | 0.92-4.16 | 0.0791 | 2.98 | 1.17-8.52 | 0.0213 |
