# Supplementary material for: Phosphorylated IκBα Predicts Poor Prognosis in Activated B-Cell Lymphoma and Its Inhibition with Thymoquinone Induces Apoptosis via ROS Release
Source: PLoS One. 2013 Mar 28;8(3):e60540. doi: 10.1371/journal.pone.0060540 (PMC3610815; doi:10.1371/journal.pone.0060540)
Supplement: Table S3 — Combination index calculation using Chou and Talalay method in ABC cell lines. HBL1 and RIVA cell lines were treated with various doses of TQ and TRAIL alone or in combination and Fraction effect (Fa), Combination Index (CI) and Dose Reduction Index (DRI) are indicated in Table S3. (DOCX) [file pone.0060540.s006.docx]

Table S3**: Combination index calculation using Chou and Talalay method in ABC cell lines.**

**---------------------------------------------HBL1-------------------------------------------------**

**Thymoquinone TRAIL Fractional Combination index Dose Reduction**

**(μM) (ng) effect (Fa) (CI) Index (DRI)**

**% Apoptosis Thiostrepton TRAIL**

**(μM) (ng)**

**0.5 11.6**

**1.0 20.0**

**5.0 48.4**

**10 65.5**

**25 84.2**

**Median dose (Dm) 4.66 μM**

**Exponent shape of curve (m) 0.92572 ± 0.034687**

**Linear correlation coefficient (r) 0.99790**

**0.5 13.6 1.0 30.3 5.0 54.5**

**10 70.0**

**25 76.4**

**Median dose (Dm) 5.744 ng**

**Exponent shape of curve (m) 1.09555 +/- 0.0095103**

**Linear correlation coefficient (r) 0.98888**

**1. 0.5 0.5 22.0 1.066 1.550 2.376**

**2. 1.0 1.0 55.0 0.359 5.358 5.792**

**3. 5.0 5.0 84.0 0.315 7.322 5.593**

**4. 10 10 77.0 1.076 2.023 1.720**

**5. 25 25 81.0 2.018 1.113 0.893**

**----------------------------------------------RIVA------------------------------------------------ Thymoquinone TRAIL Fractional Combination index Dose Reduction**

**(μM) (ng) effect (Fa) (CI) Index (DRI)**

**% Apoptosis** **Thiostrepton TRAIL**

**(μM) (ng)**

**0.5 17.1**

**1.0 18.7**

**5.0 58.5**

**10 65.3**

**25 76.4**

**Median dose (Dm) 4.57 μM**

**Exponent shape of curve (m) 0.76934 +/- 0.079360**

**Linear correlation coefficient (r) 0.98441**

**0.5 19.0**

**1.0 36.3**

**5.0 43.2**

**10 69.4**

**25 80.0**

**Median dose (Dm) 3.70 ng**

**Exponent shape of curve (m) 0.67064 +/- 0.108721**

**Linear correlation coefficient (r) 0.96277**

**1. 0.5 0.5 26.0 1.068 3.604 7.159**

**2. 1.0 1.0 68.2 0.168 11.437 49.296**

**3. 5.0 5.0 67.1 0.900 2.681 12.351**

**4. 10 10 83.7 0.496 1.412 6.649**

**5. 25 25 90.0 0.569 0.876 4.957**
